# Current Challenges in Plant Cell Walls: Editorial Overview

**DOI:** 10.3389/fpls.2012.00232

**Published:** 2012-10-17

**Authors:** Seth DeBolt, Jose M. Estevez

**Affiliations:** ^1^Department of Horticulture, University of KentuckyLexington, KY, USA; ^2^Laboratorio de Fisiología y Biología Molecular, University of Buenos Aires and Consejo Nacional de Investigaciones Científicas y Técnicas, IFIByNEBuenos aires, Argentina

## Expanding Methodologies and Models to Study the Plant Cell Walls

An exciting feature of this special issue was the injection of a series of papers dealing with developed and developing methodological approaches applied to cell wall biosynthesis. For example, our understanding of cell wall synthesis and remodeling has been limited by a lack of suitable chemical probes that are compatible with live cell imaging. Here, Wallace and Anderson (2012) summarize the currently available molecular tool box of probes available for cell wall polysaccharide imaging in plants, with particular emphasis on recent advances in small molecule-based fluorescent probes such as the cellulose-binding dyes S4B and xyloglucan-binding dye 7GFE. In addition, these authors investigated the potential of “click” chemistry to label specific cell wall polysaccharides. The term “Click” chemistry describes chemistry tailored to generate substances quickly and reliably by joining small units together. They explore selectivity issues and highlight how structure – activity relationships of click sugar variants may be used to elucidate cell wall structural associations. Gonneau et al. (2012) carried on with live cell reporter theme and explored a large panel of fluorescent imaging techniques and reporters currently available for the study of tissues, cells, and cellular components in three dimensions (3D) or even in 4D (3D + time) in plants. These imaging techniques hold promise to greatly improve our ability to ask cause and effect questions regarding the dynamic construction and deconstruction of cell wall architecture.

*N*-glycosylation is one of the most common and complex posttranslational modifications of secreted eukaryotic proteins. Yet in plants, the functional significance of *N*-glycosylation has been poorly explored and lags far behind other eukaryotic models. Ruiz-May et al. (2012a) provide a seminal review on the range of existing analytical technologies for large-scale *N*-glycoprotein analysis. This review provides insight into the myriad of hurdles one may expect during glycopeptide amino acid sequencing by mass spectral approaches. In turn, the authors present a step-by-step guide to selective enrichment, improved analytical platforms, and software that can be used to wade through MS fragmentation data. This framework is then employed in a review of plant *N*-glycoprotein synthesis and trafficking (Ruiz-May et al., 2012b), which exemplifies those proteins that are cell wall localized. Thus, the contribution of Ruiz-May and coworkers provides a targeted resource for researchers who meet a cell wall localized glycoprotein. Bauer (2012) then provides a review of MS analytical techniques to identify structural information about complex carbohydrate (non-protein) composition of the plant cell wall. Here, powerful MS methods are detailed for the elucidation of the structure of oligosaccharides derived from hemicelluloses and pectins. The author illustrates how information on sequence, linkages, branching, and modifications can be obtained from characteristic fragmentation patterns. This method is of further note when combined with molecular genetics (mutant analysis) to elucidate the function of a target gene.

Global transcript analyses based on a wealth of available microarray datasets have revealed that genes that are in the same pathway are often transcribed in a coordinate manner. Here Ruprecht and Persson (2012) have summarized recent developments in software as well as combining transcriptional data from multiple plant species to learn about how particular cell wall processes are different, rather than the same. This exciting update provides successful examples of where expression profiling has helped to identify genes involved in the formation of cellulose, hemicelluloses, and lignin. In addition, they illustrate potential pitfalls and future perspectives. The broad employment of next generation sequencing for RNAseq experiments will surely expand the available data (as not all coding sequences are represented on a given microarray chip). This will require some software refinement, but also see further gains resulting from coexpression profiling in the near future. The use of deep sequencing platforms like Illumina’s Genome Analyzer or ABI’s SOLiD was reviewed by Vidaurre and Bonetta (2012) in the context of map based cloning. Here, a path to rapidly accelerating forward genetics in the context of cell wall biosynthesis and deconstruction was paved. Anyone who has laboriously mapped a missense mutation will greatly appreciate the ability to pinpoint a mutation selected from within an EMS (ethyl methyl suflonate) population in a single sequencing run. This combination of a classical genetics approach and cutting edge technology can holds immediate promise for gene identification.

To assemble plant cell wall polysaccharides and glycan structures on glycoproteins, the plant needs extensive biosynthetic machinery, and it has been estimated that over 2000 gene products are involved in making and maintaining the wall. The Carbohydrate Active Enzyme (CAZy) database is an invaluable resource for glycobiology and currently contains 45 glycosyltransferase (GT) families that are represented in plants. Hansen et al. (2012) describe the putative GTs that are not currently classified in the CAZy database. These families include proteins with domain of unknown function (DUF). The evidence for certain members of the DUF class proteins being GTs and their possible roles in cell wall biosynthesis are discussed.

Model organisms have been instrumental in fundamental discovery based science over the past several decades. A model for grass cell wall biology has lagged behind its dicotyledonous counterpart, *Arabidopsis*. Handakumbura and Hazen (2012) provide a review of the transcriptional wiring of secondary cell wall biosynthesis in grasses. Many grasses have abundant sequence information including Corn (*Zea mays* L.), barley (*Hordeum vulgare* L.), and rice (*Oryza sativa* L.) and currently *Brachypodium distachyon* is emerging as a model for functional genomics and gene discovery. Handakumbura and Hazen (2012) focus on NAC and MYB transcription factors and review methods for establishing functionality of these regulatory genes. Characterization of the transcriptional regulatory networks of eudicots and grasses remains of high priority in efforts to optimize biomass quality. Perhaps better still than a model organism, is a model cell type in order to genetically decode a target process. Haigler et al. (2012) and Haughn and Western (2012) examine this notion using cotton fibers and *Arabidopsis* seed coats respectively. Both cell types display intriguing polarized differentiation characteristics and represent models to study the role of cell walls in morphogenesis as well as general cell wall biosynthesis. The *Arabidopsis* seed coat differentiation involves the deposition of a large pectin rich mucilage pocket on the outer side of the seed coat (Haughn and Western, 2012). Therefore, the cell type lends itself to investigating the chemical and physical properties of different cell wall components and their interactions *in vivo*, especially with respect to its major pectic component rhamnogalacturonan I (RGI). Similarly, cotton fibers are single-celled extensions of the seed epidermis. They can be isolated in pure form as they undergo staged differentiation including primary cell wall synthesis during elongation and nearly pure cellulose synthesis during secondary wall thickening. Haigler et al. (2012) exemplify the potential to combine virus induced gene silencing as a genetic tool with systematically staging of developmental phenotypes to provide a highly relevant tool to dissect open questions in cellulose biosynthesis.

## Concepts in Cell Wall Biology

Plant cell walls provide structural support during development and represent the first line of defense against biotic and abiotic stress. In recent years evidence has accumulated that a dedicated plant cell wall integrity maintenance mechanism exists. Here, Hamann (2012) reviewed the available evidence regarding the mode of action of the cell wall integrity maintenance mechanism and discuss its role in the context of biotic plant stress response mechanisms. Recent strides in our understanding of surface defense or cell wall-associated defenses induced by pathogen perception have been made. Underwood (2012) reviews these in context of the impact of these changes in cell wall polymers and highlights significant unanswered questions driving future research in the area. These include plasma membrane-cell wall adhesion, a critical site for pathogen recognition and signal transduction. Along this theme, the wall-associated kinases (WAKs, receptor-like kinases) that associate with pectin in the cell wall and contain a cytoplasmic kinase domain may play a role in such a transducing signal on wall development and or pathogen ingress. Indeed, Kohorn and Kohorn (2012) delve into the world of WAKs and investigate a role in receptor sensing for both short oligogalacturonic acid fragments generated during pathogen exposure or wounding, and longer pectins resident in plant cell wall biosynthesis. The authors look into the capability of WAKs to bind and respond to several types of pectins and highlight the many remaining unanswered questions.

The plasma membrane-cell wall continuum also appears to require a membrane organizational matrix. Schrick et al. (2012) highlights the possible direct or indirect involvement of membrane structural sterols in facilitating the cellulose biosynthetic machinery. In one possible scenario, molecular interactions in sterol-rich plasma membrane microdomains or another form of sterol-dependent membrane scaffolding may be needed for correct subcellular localization, structural integrity, and/or activity of the cellulose synthase (CESA) machinery. In this context, Davis (2012) takes a biochemical view and rationalizes how polymers requires transport across a membrane and many polysaccharide biosynthetic enzymes appear to have both transporter and transferase activities. Drawing on what is known about such dual-function enzymes in other species, the author examines the required number of transmembrane helices, phosphorylation regulation, and the importance of the membrane environment surrounding the enzyme. This review does an exceptional job of highlighting where deficiencies reside in current models and sets up many questions for immediate elucidation. Of course, many cell wall enzymes modify the structurally diverse polymers of the cell wall, an example being the elusive enzymes responsible for extensive *O*-acetylation. Exactly how plants *O*-acetylate wall polymers and why has remained elusive until recently, when two protein families were identified in the model plant *Arabidopsis* that are involved in the *O*-acetylation of wall polysaccharides. Gille and Pauly (2012) provide a detailed review discussing the role of these two protein families in polysaccharide *O*-acetylation and outlines the differences and similarities of polymer acetylation mechanisms in plants, fungi, bacteria, and mammals.

Dropping beneath (literally) the plasma membrane-cell wall continuum, both the actin and microtubule cytoskeleton are involved in assuring the proper distribution, organization, and dynamics of CESA complexes (CSCs). The review of Lei et al. (2012) provides an update on the characterization of the composition, regulation, and trafficking of CSCs and also introduces a newly identified CESA interactive protein1 (CSI1). The CESA superfamily of proteins contains several sub-families of closely related CESA-LIKE (CSL) sequences. Among these, the CSLA and CSLC families are closely related to each other and are the most evolutionarily divergent from the CESA family. Liepman and Cavalier (2012) summarize some of the most significant progress that has been made with the functional characterization of CSLA and CSLC genes, which have been shown to encode enzymes with 1,4-β-glycan synthase activities involved in the biosynthesis of mannan and possibly xyloglucan backbones, respectively. The authors highlight a series of key questions, whose answers likely will provide further insight about the specific functions of members of the CSLA/C families.

Plant cell walls are not only composed by large amounts and diverse types of polysaccharides but also *N*- and *O*-glycoproteins. Within *O*-glycoproteins, Arabinogalactan-glycoproteins (AGPs) and Extensins (EXTs) are commonly glycosylated. The genetic set up and the enzymes that define the *O*-glycosylation sites and transfer the activated sugars to cell wall glycoprotein EXTs have remained unknown for a long time. Velasquez et al. (2012) summarizes several of the genes recently discovered that confers the posttranslational modifications, i.e., proline hydroxylation and subsequent *O*-glycosylation, of the EXTs. The effects of posttranslational modifications on the structure and function of EXTs in plant cell walls is also discussed. The location and diversity of AGPs have made them attractive targets as modulators of plant development but definitive proof of their direct role(s) in biological processes remains elusive. Tan et al. (2012) further reviews the current state of knowledge on AGPs. These authors identify key challenges impeding progress in the field and propose approaches using modern bioinformatic, (bio)chemical, cell biological, molecular, and genetic techniques that could be applied to address these conceptual gaps.

Pectins are one of the most complex polymers within the plant cell wall. Bar-Peled et al. (2012) draws attention to the evidence showing that the pectic polysaccharide rhamnogalacturonan II (RG-II) exists in the primary cell wall as a borate cross-linked dimer and that this dimer is required for the assembly of a functional wall and for normal plant growth and development. RG-II structure and crosslinking is well conserved in vascular plants and that RG-II likely appeared early in the evolution of land plants. Although many of the genes involved in the generation of the nucleotide sugars used for RG-II synthesis have been functionally characterized, only one GT involved in the assembly of RG-II has been identified. In addition, these authors provide an overview of the formation of the activated sugars required for RG-II synthesis and point to the possible cellular and metabolic processes that could be involved in assembling and controlling the formation of a borate cross-linked RG-II molecule. They also discuss how nucleotide sugar synthesis is compartmentalized and how this may control the flux of precursors to facilitate and regulate the formation of RG-II. Still focusing on pectin, Peaucelle et al. (2012) provide a compelling investigation into the role of pectin in cell wall mechanics. Specifically, in the past most studies have focused on the role of the cellulose/xyloglucan network (reviewed in Zabotina, 2012) and the enigmatic wall-loosening agents expansins. By contrast, cell wall synthesis in the land plants close evolutionary progenitor, the Charophycean algae, is coupled to cell wall extensibility by a chemical Ca^2+^-exchange mechanism between Ca^2+^–pectate complexes. In land plants, this mechanism would provide an intriguing alternative. Authors review the current evidence for the existence in terrestrial plants of a “primitive” Ca^2+^-pectate-based growth control mechanism in parallel to the more recent, land plant-specific, expansin-dependent process.

Plant cell walls are vey dynamic and change in both structure and composition over time, cell development, and differentiation, environmental conditions, etc. On the other hand, despite differences in composition and structure between dicots and monocots, all flowering plants respond to a defined suite of growth regulators in similar tissue-specific ways and also exhibit similar growth physics. Using this rationale, Benatti et al. (2012) examine the physiological control of cell expansion emerging from genetic functional analyses, mostly in *Arabidopsis* and other dicots, and a few examples of genes of potential functions in grass species. These authors discuss examples of cell wall architectural features that impact growth, independent of composition, and progress in identifying proteins involved in transduction of growth signals, and the integration of their outputs in the molecular machinery of wall expansion. In another insightful minireview, Cosgrove and Jarvis (2012) discuss the essential differences between primary and secondary cell walls and identify crucial gaps in our knowledge of their structure and biomechanics. The authors provide compelling evidence that there is a particular need to revise and correct current “cartoon depictions” of plant cell walls, so that they are more consistent with present data available from diverse approaches.

## Plant Cell Walls: Structural Diversity and Evolutionary Aspects

While all of the contributions in this special issue provide important insights into cell walls that ultimately fit into an evolutionary context, three reviews attempt to piece together cell wall evolutionary processes (Domozych et al., 2012; Fangel et al., 2012; Roberts et al., 2012). The first examines the green algae, which represent a large group of morphologically diverse photosynthetic eukaryotes that display much diversity in their cell walls. They compare and contrast the Charophycean green algae, which possess cell walls compositionally similar embryophyte walls with that of the Ulvophycean seaweeds, which have cell wall components whose most abundant fibrillar constituents may change from cellulose to β-mannans to β-xylans and during different life cycle phases. Cell wall composition is incorporation of emerging genomic and transcriptomic data to divulge evolutionary trends. The second review grapples with the diversity of plant cell wall structures. An additional review by Roberts et al. (2012) presents the molecular and physiological features of cell wall biosynthesis of the moss *Physcomitrella patens*. The cell walls of mosses and vascular plants are composed of the same classes of polysaccharides as found in moss, but with differences inside chain composition and structure. Similarly, the genomes of *P. patens* and angiosperms encode the same families of cell wall GTs, yet, in many cases these families have diversified independently in each lineage. Combined, these three reviews capture the spectrum of cell wall evolutionary processes and highlight numerous underexplored cell walls types and processes that are underexplored and could provide a rational starting point in isolating industrially useful bioproducts.

As a concluding remark, we are incredibly grateful to the all of the contributors including authors, reviewers, and the *Frontiers* editorial office for their generous willingness to participate in this effort.
